# Phenotypic and Genotypic Characterization of *C. perfringens* Isolates from Dairy Cows with a Pathological Puerperium

**DOI:** 10.3390/vetsci9040173

**Published:** 2022-04-04

**Authors:** Hanna Kronfeld, Nicole Kemper, Christina S. Hölzel

**Affiliations:** 1 Department for Animal Hygiene and Animal Health, Institute of Breeding and Husbandry, Christian-Albrechts-University, Olshausenstr. 40, 24098 Kiel, Germany; choelzel@tierzucht.uni-kiel.de; 2 Institute for Animal Hygiene, Animal Welfare and Farm Animal Behaviour, University of Veterinary Medicine Hannover, Foundation, Bischofsholer Damm 15, 30173 Hannover, Germany; nicole.kemper@tiho-hannover.de

**Keywords:** *Clostridium perfringens*, dairy cows, metritis, uterine infection, puerperium

## Abstract

*Clostridium perfringens* (*C. perfringens*) forms part of the intestinal microbiome, but is also a known pathogen in histotoxic infections. The significance of the pathogen as a cause of uterine infections in cattle has been little studied so far. Here, we analyzed the association between a pathological puerperium in cattle and the detection of *C. perfringens* in a prospective longitudinal study. *Clostridium perfringens* were only found in vaginal and uterine samples of diseased cattle, and were absent in healthy controls. Isolates (*n* = 21) were tested for the production of major toxins (alpha-, beta-, epsilon-toxin) by ELISA and for the potential of production of major (alpha-, beta-, iota-toxin) and minor toxins (beta2 toxin) by PCR. Furthermore, antimicrobial susceptibility was also tested phenotypically by microdilution. Despite the frequent use of tetracycline treatment in cows suffering from puerperal disorders, no isolate showed phenotypic tetracycline resistance. Most isolates did not release major amounts of toxin. The strict association of *C. perfringens* with puerperal disease, together with the absence of major toxins might hint towards a major role of other or unknown clostridial virulence factors in uterine disease.

## 1. Introduction

*Clostridium perfringens* (*C. perfringens*) is primarily known as an enteropathogenic bacterium and as a causative agent of enterotoxaemia [1,2]. The bacterium also has histotoxic potential, the best-known clinical picture being that of gas gangrene [1,2,3]. The pathogenicity of *C. perfringens* is based in particular on its ability to produce toxins; a distinction is made between major toxins and minor toxins. Based on the major toxins (α, β, ε, ι), *C. perfringens* is divided into five types (A, B, C, D, E); see Table 1 [1,2]. The alpha toxin is a phospholipase C and attacks cell membranes leading to cell lysis. This also results in a dermonecrotic and haemolytic effect [4]. Furthermore, a vasoconstrictive effect has been described [5]. The *cpa* gene, which codes for the alpha toxin, is chromosomally localized, unlike most other toxins included in this study. Thus, *cpa* is found in all *C. perfringens* strains [6,7,8]. However, the strains of type A usually produce larger amounts of toxin A than the other types [9]. The beta toxin is encoded by the plasmid-borne *cpb1* gene [7] and leads to pore formation in membranes, which results in lysis [10]. This cytotoxic activity also results in dermonecrotic and enterotoxic effects. The toxin can bind to endothelial cells, which can lead to degeneration, thrombosis and necrosis [5]. The *etx* gene is also plasmid-borne and encodes the epsilon toxin, a pore-forming toxin causing cell death [11] and attacking vascular endothelia, which can lead to oedema [1,7]. Iota toxin, an actin-ADP-ribosylating toxin, is a binary toxin with a binding and an enzyme component. Both components are necessary for the biological effects [8,12], and the genes (*iap* and *iab*) are also encoded on plasmids [8].

The minor toxins include the beta2 toxin (*cpb2* gene), which has so far been associated primarily with enteritis, and the theta toxin (perfringolysin O; *pfoA* gene, chromosomally localized), which is associated in particular with wound infections [7,13].

There are few published data linking *C. perfringens* to reproductive tract diseases in farm animals [14,15]. Our data provided note for the involvement of the species in the pathogenesis of typical puerperal diseases in dairy cows. Usually, inflammation of the uterus is treated with antibiotics [16]. In particular, penicillin, cephalosporins and tetracyclines are used [16,17]. Any use of antibiotics can contribute to the selection of resistant bacteria [18].

Thus, the aim of our study was to specify the role of *C. perfringens* in causing uterine infections and to genotypically and phenotypically characterize 22 *C. perfringens* isolates from the vagina and uterus of cows with puerperal disorders.

## 2. Materials and Methods

### 2.1. Clinical Gynaecological Examination and Sampling

Vaginal and uterine swab samples were collected from 46 pluriparous dairy cows of one herd at 4 time points (day 2, 4, 6 and 14 p.p.) as part of a clinical gynaecological examination. On day 22 ± 1 and 41 ± 1, the dairy cows were clinically re-examined to re-evaluate the health status. In addition, a birth history report was recorded for each animal, including antibiotic treatments. 

As part of the clinical investigation, the rectal body temperature was measured (Microlife^®^ VT 1831, Microlife AG, Widnau, Switzerland). The vaginal discharge was classified by vaginoscopy. Furthermore, the degree of moisture and colour of the vaginal mucosa and the portio vaginalis, the shape and the degree of opening of the portio vaginalis were assessed. Based on the clinical gynecological examination, the cows were categorized as healthy or diseased (postpartum retention, metritis, or clinical endometritis). 

The collection of the vaginal swab was performed before vaginoscopy. The external genitalia was washed with iodine-containing soap (Iodosept^®^PVP, Vetoquinol GmBH, Ismaning, Germany) and dried with a paper towel. The labia were spread, the sterile cotton headed swab (cotton stick, Boettger, Bodenmais, Germany) inserted and rotated on the vaginal roof for over 10 s. After vaginoscopy, the uterine swab were taken. The cervix was grasped rectally and carefully pulled cranially, the sterile double-protected swab (uterine culture swab Minitube, Tiefenbach, Germany) was inserted into the uterus and rotated for 10 s on the endometrium of the uterine roof. The samples were transferred to a transport medium (Amies Transport Medium, Thermo Scientific TM, Schwerte, Germany) and brought to the laboratory within 12 h while maintaining a cold chain.

### 2.2. Antibiotic Treatment

The antimicrobial treatment was prescribed and administered by the farm’s attending veterinarian. The treatment was independent of the participation in the study and was recorded retrospectively. Cows with a pathological puerperium without fever were treated locally with antibiotic uterine sticks (6000 mg tetracycline hydrochloride) three times at intervals of two days, and those with fever were additionally treated with systemic antibiotics (7500 mg benzylpenicillin procaine) twice at intervals of 24 h.

### 2.3. Bacteriological Cultures

Samples were thoroughly vortexed, and dilution series (10^−1^ to 10^−3^) were prepared. Samples were plated onto Schaedler agar (37 °C, 48 h, anaerobic; Thermo ScientificTM, Schwerte, Germany). All morphologically different bacteria were subcultured, identified by MALDI-TOF-MS (Bruker, Billerica, MA, USA) and cryopreserved at −80 °C. The number of (facultative) anaerobic bacteria was recorded semi-quantitatively per swab. For further investigations, the *C. perfringens* pure cultures were subcultured on Columbia agar with 7% sheep blood (37 °C, 24 h, anaerobic).

### 2.4. ELISA

For the detection of alpha-, beta- and epsilon toxins, the *C. perfringens* isolates were subcultured and culture supernatants were prepared. To obtain the culture supernatants, small amounts of the *C. perfringens* subcultures were transferred into freshly prepared TGY medium. The tubes were incubated anaerobically for 4 h (alpha toxin) or overnight (epsilon- and beta toxin) at 37 °C without shaking. An enzyme-linked immunosorbent assay (ELISA; BIO K 270/2, Bio-X Diagnostics S.A., Rochefort, Belgium) was used to detect the production of toxins potentially contained in the culture supernatants. The culture supernatants (100 µL) were pipetted undiluted into the wells and the covered sample plate was incubated at 21 °C for 1 h. After washing three times (300 µL of wash buffer per well and wash), 100 µL of the conjugates (peroxidase-labelled, monoclonal or polyclonal antibodies) were added per well. The microtitre plate was covered and incubated again at 21 °C for 1. Afterwards, the plate was again subjected to three washes. Then, 100 µL of the developer solution (Tetramethylbenzidine colour solution) was pipetted into the wells. The plate was incubated for 10 min at 21 °C without cover, but protected from light. Finally, 40 µL of the stop solution (Phosphoric acid) was added per well and the optical density was immediately measured with a photometer (BioPhotometer D30, Eppendorf, Hamburg, Germany) at 450 nm. Every second row of the microtitre plate was coated with non-specific antibodies and served as a negative control; the positive control was included in the first column. The measured optical densities of the negative control wells were subtracted from those read on the corresponding wells coated with specific antibodies, resulting in delta OD-values representing the difference in absorbance. Finally, each delta OD-value of a sample was divided by the delta OD-value of the corresponding positive control antigen, and the result was multiplied by 100 so that it could be given as a percentage.

### 2.5. Bacterial Reference Strains

*Clostridium perfringens* DSM 756 (type A), CCUG 2035 (type B), CCUG 44727 (type E) and CCUG 42881 (beta2) were used as positive controls for the toxin genes detection. Type B was used as genomic DNA. *Clostridium perfringens* DSM 756 was used as quality control for microdilution.

### 2.6. DNA- Extraction

The *C. perfringens* isolates and reference strains were subcultured on Columbia agar with 7% sheep blood (24 h, 37 °C, anaerobic). A small amount of bacteria was removed from the agar plate with an inoculation loop and suspended in 180 µL of buffer ATL. Deoxyribonucleic acid was extracted according to manufacturer’s instructions following the extraction protocol for isolation of genomic DNA from Gram-positive bacteria (QIAmp DNA minikit, Qiagen, Hilden, Germany). The elution was stored at −20 °C.

### 2.7. Detection of Toxin Genes by PCR

Genotyping was performed by PCR amplification of three major toxins (alpha-, beta- and iota toxin) and one minor toxin (beta2) following the protocol of Gkiourtzidis et al. [19]. The PCR was carried out in a thermocycler (FlexCycler, Analytic Jena, Jena, Germany). The total volume of the reaction was 50 µL, containing 25 µL mastermix (ReadyMix^TM^Taq PCR Reaction Mix with MgCl_2_, sigma-aldrich, St. Louis, MI, USA), 22 µL nuclease free water, 2 µL primer (Table 2) and 1 µL template-DNA. The PCR comprised 35 cycles; the first PCR cycle consisted of a 5 min denaturation at 94 °C, with the following consisting of 5 min 30 s denaturation at 94 °C, 30 s annealing at different temperatures (*cpa*: 46 °C, *cpb1:* 39 °C, *iap:* 46 °C, *cpb2:* 48 °C) and 30 s of extension at 72 °C and a subsequent extension of 5 min also at 72 °C. The primers used are listed in Table 2. Subsequently, the PCR products were visualized by gel electrophoresis.

### 2.8. Antimicrobial Susceptibility Testing

Phenotypic detection of antibiotic resistance was performed by the broth microdilution method (MICRONAUT-S Anaerobes MIC, Merlin Diagnostika GmBH, Bornheim-Hersel, Germany). The plate configuration includes 13 dehydrated antibiotics: amoxicillin/clavulanate, ampicillin, clindamycin, doxycycline, ertapenem, imipenem, meropenem, metronidazole, moxifloxacin, penicillin G, piperacillin/tazobactam, tigecycline and vancomycin. A 0.5 McFarland bacterial suspension with NaCl (0.9%) was prepared from all *C. perfringens* isolates, 200 µL of the bacterial suspension was transferred into 11.5 mL ready-to-use tubes with Wilkens-Chalgren broth (MICRONAUT-Wilkens-Chalgren broth, Merlin Diagnostika GmBH, Bornheim-Hersel, Germany), which had previously been pre-tempered (37 °C) under anaerobic conditions for 90 min, and the MICRONAUT-S Anaerobes MIC plate was inoculated with 100 µL of the suspension and incubated anaerobically at 37 °C for 24 h. Finally, the test plates were read visually and evaluated according to the CLSI- standard (M100, 31st edition, March 2021).

### 2.9. Statistical Analysis

The statistical analysis and graphical processing was performed with GraphPad Prism (version 9.3.1; GraphPad Software, San Diego, CA, USA). The Fishers exact test was used to test whether significantly more diseased cows were positive for *C. perfringens*. Furthermore, the Fisher’s exact test was used to compare whether significantly more cows were *C. perfringens*-positive on day 2 p.p. than on day 4 p.p. In addition, the Spearman rank test was used to test whether there was a correlation between the presence of *C. perfringens* in the vagina and the presence of the species in the uterus, for which purpose the correlation coefficient (ρ) was declared.

## 3. Results

**Figure 1:**
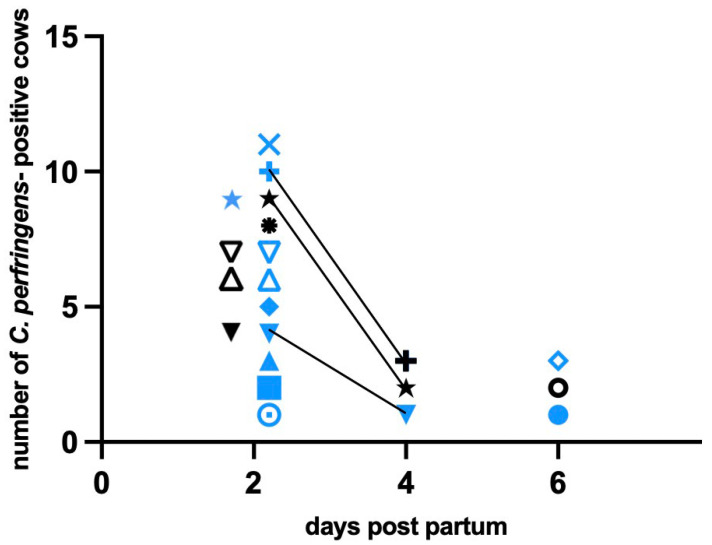
Visualization of the distribution of *C. perfringens* isolates (*n* = 21) among the hosts (*n* = 14) in relation to the time of sampling and the localization; each symbol stands for an isolate, different symbols symbolize the cows, different colors symbolize the localization; black = vagina, blue = uterus, line = follow-up isolate.

**Figure 2:**
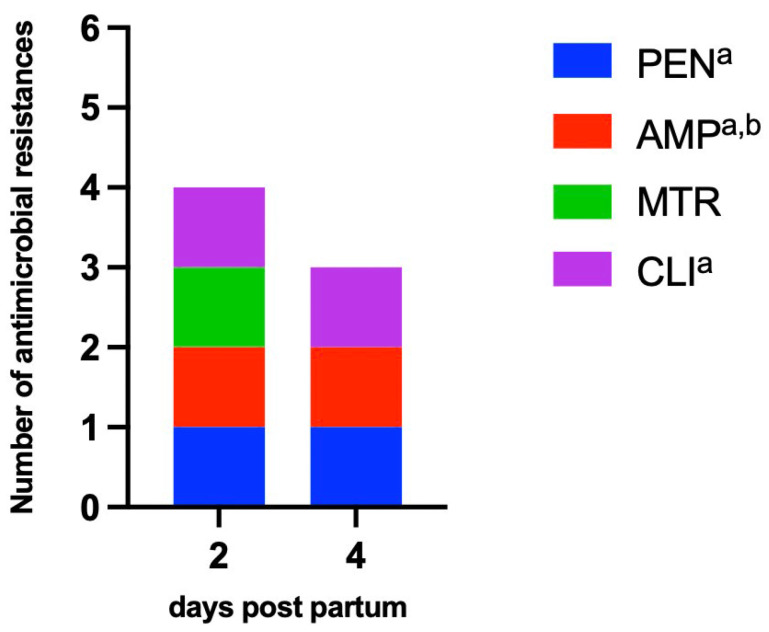
Sum of antimicrobial resistances compared among the sampling dates, *C. perfringens* isolates *n* = 3 (day 2 *n* = 2; day 4 *n* = 1) from two antibiotic- treated cows; PEN = penicillin, AMP = ampicillin, MTR = metronidazole, CLI = clindamycine; a = on day 2 and day 4 p.p., one isolate each could be detected from the same animal, b = AMP on day 4 intermediate; one cow was treated with benzylpenicillin procaine, the other cow additionally with tetracycline hydrochloride and Penethamathydroiodid (due to additional mastitis), treatment start for both cows: day of calving.

Based on clinical gynaecological examination, 27 cows out of 46 animals showed a pathological puerperium. At present, the microbiological results of 12 healthy and all diseased animals are available. *Clostridium perfringens* could not be isolated from any of the healthy cows, but 14 of the diseased animals were positive for *C. perfringens* (51.9%). Table 3 lists the clinical pictures of the 14 *C. perfringens* positive animals; it should be noted that clinical pictures can merge into one another. Significantly more cows were positive on day 2 p.p., while a minority of cattle were positive on day 4 and 6 p.p. (Fishers exact test *P* 0.007), and more cows were positive in the uterus (Table 4, Figure 1). A significant correlation between the occurrence of the species in the vagina and uterus could not be established, but the occurrence of the species tended to increase together (Spearman correlation; rho = 0.321). Based on the ELISA-results, the production of alpha toxins could be phenotypically proven in 3 isolates. The *cpa* gene was detected in all 21 isolates by PCR. The toxin gene *iap* could not be detected, the *cpb1* gene in two isolates and the *cpb2* gene could be detected in one isolate. With regards to the phenotypic antimicrobial susceptibility, 3 isolates showed resistance. Against penicillin G and clindamycine, two isolates showed resistance (Table 5 and Table 6). Resistance to ampicillin and metronidazole was also detected. The antimicrobial resistant three *C. perfringens* isolates came from two antibiotic-treated cows. In one case, the isolates were consecutive: two isolates were isolated from the uterus of one cow on days 2 and 4 p.p. and showed the same resistance profiles, except that the isolate from day 4 p.p. did not show clinical ampicillin resistance, but was classified as intermediate. No antibiotic-resistant *C. perfringens* could be isolated on day 6 and 14 p.p. anymore; see Figure 2. Two isolates of a treated cow showed resistance to 3 resp. 2 antibiotics, including penicillin G, with which the cow was treated (Figure 2).

## 4. Discussion

This study found an exclusive occurrence of *C. perfringens* in cattle with a pathological puerperium compared to healthy animals on a farm with high infection rates (27/46). Of the diseased animals, half were *C. perfringens* positive (51.9%), compared to 0 of 12 healthy animals. Due to the fact that we isolated all culturable bacteria from the samples, identification was very time-consuming and could be done only for a subset of the healthy animals (12/19); however, the results are statistically significant (fishers exact test *p =* 0.02). So far, the species *C. perfringens* has mainly been associated with intestinal diseases [23]. However, there are also reports of diseases of other body sites caused by *C. perfringens*, such as the udder [15,24]. Findings on diseases of the reproductive tract caused by this pathogen are rare. Klein et al. [14] report purulent ulcerative vestibulovaginitis and myometritis in highly gravid sheep caused by *C. perfringens*. From human medicine, there are also reports of *C. perfringens* as a causative agent of uterine infections [25,26,27,28]. The clinical pictures vary from uncomplicated endometritis to gas gangrene and fulminant septicaemia [25]; the authors point out that the pathogen is a rare but feared cause of uterine infection [25,26,27,28]. Wang et al. [29] describe *C. perfringens* as a common species in the postpartum vagina of cattle and were able to isolate *C. perfringens* in two healthy (of total *n* = 5) cows and one endometritic (of total *n* = 5) cow. Other authors also asses *C. perfringens* as a contaminant with regard to pathogenic uterine potential [30]. There is no doubt that *C. perfringens* as an intestinal inhabitant might contaminate vaginal swabs. However, with our sampling method giving high regard to avoiding contamination (by washing the vulva and using double-protected swabs), we did not find *C. perfringens* in any of the healthy cows, although we sampled a higher number, compared to Wang et al. [29]. According to a conference contribution, Dunaiev et al. [15] were also able to detect *C. perfringens* in 46.9% (*n* = 23) of endometritic cattle, with deficiencies in farm management, including hygiene and treatment management, found on the study farms. However, no comparison with healthy animals was made there, and up to now, representative investigations in cattle have been lacking. In our study, not only the exclusive occurrence in healthy cattle, but the localization and time of detection support the assumption that *C. perfringens* has a biological significance in the pathogenesis of uterine diseases, since *C. perfringens* occurred more frequently in the uterus and significantly more cows were positive on day 2 p.p., with or shortly before the onset of symptoms. The determination of the semi-quantitative number of (facultative) anaerobes per swab showed that *C. perfringens* often plays a dominant role (range 1–6, median: 2). Other potential uterine pathogens were also detected in *C. perfringens* positive animals, but accompanying bacterial profiles showed a lack of uniformity (Table 7). *Escherichia coli* and *Trueperella pyogenes* are frequently mentioned in literature as pathogens for uterine infections [30], but in our study lot, the two species were not significantly elevated in cows with a pathological puerperium (healthy *n* = 12, diseased *n* = 10) as found in a separate study on the postpartum bovine vaginal and uterine microbiome. Thus, in our dataset only *Fusobacterium* spp. was co-associated with disease, but co-occurred in only 8 of 14 *C. perfringens*-positive cattle.

Postpartum (micro-)lesions, a low-oxygen environment in utero and injured endometrium [14] and an excellent culture medium in the form of the lochia provide *C. perfringens* with good conditions to multiply and establish. Assuming that *C. perfringens* plays a biological role in etiopathogenesis, the toxin production, the genetic potential of toxin production and the amount of clostridia have to be considered [31], taking into account the competition of other bacteria and the immune response of the host. To verify the hypothesis that *C. perfringens* is a causative agent in uterine disease, toxin production and genetic potential of toxin production were investigated. Three isolates could be phenotypically confirmed as alpha toxin producers, but all isolates showed the potential of production. There is a divergence between phenotypic production and the corresponding genotypic potential, which is why it makes sense to look at and evaluate both sides in parallel. Unfortunately, we did not have functioning controls of all relevant clostridial virulence factors available. However, we assessed three major virulence factors by ELISA (alpha, beta, epsilon toxin) and two of them also by PCR (*cpa, cpb1*), together with the minor toxin gene *cpb2.* In vitro, most of the isolates did not produce major amounts of toxins, except three isolates from three cows with clinical metritis and postpartum retention of the fetal membranes; two of the cows showed puerperal metritis (grade 1 and 2; *n* = 1 each), whereby the inflammation of the cow with grade 1 puerperal metritis developed into clinical endometritis. For other isolates from cows with severe clinical signs we could not prove the presence of *cpb2* or production of alpha toxins. However, in vitro results must not necessarily resemble the situation in vivo. A modulation of virulence gene expression by environmental conditions is known, and good nutrient and growth conditions promote virulence gene expression in *C. perfringens* [32]. Low environmental pH has a negative effect on alpha toxin production without affecting the biomass of *C. perfringens* [33]. In addition, other virulence factors, which could not be assessed in our study or might even be unknown up to now, might play a role in the pathogenesis of uterine disease.

Uterine infections often result in antibiotic treatment that potentially selects bacterial antibiotic resistance. In the study farm, most cattle with a pathological puerperium were treated with tetracycline and/ or penicillin (63.0%). Penicillin and beta-lactam with beta-lactamase inhibitor are considered antibiotics with very good antimicrobial activity against *C. perfringens*, whereas the tetracyclines, with the exception of tigecycline, are considered to be less effective [34]. Osman et al. [35] also report a prevalence of ampicillin resistance of 7% for *C. perfringens* isolates from broiler chickens. For doxycycline resistance, the authors report a much higher frequency of 98%, which might be connected to the frequent use of antibiotics in the poultry sector, which is well known [36]. However, 70.6% of the sick and treated cows in the study farm also received tetracycline. Despite the fact that most animals were treated with antibiotics, the prevalence of penicillin resistance of 9.5% for penicillin G and 4.8% for ampicillin in the vaginal and uterine isolates is comparatively low (Table 5). We would like to point out that we did not use veterinary-specific breakpoints. However, we did not see indication for wildtype-splitting breakpoints, since MICs of resistant isolates multiplied the MICs of sensitive strains at least fourfold. The animals with resistant *C. perfringens* isolates had both already been under antibiotic treatment for 2 days. Both cattle were treated with benzylpenicillin, to which *C. perfringens* showed resistance in one of the two cattle. This illustrates that, in such cases, selection of resistant bacteria must be expected.

## 5. Conclusions

In this study, *C. perfringens* of dairy cows was associated for the first time with pathological puerperium in a prospective study providing a healthy control group. In order to correctly assess *C. perfringens* positive findings and its influence in the etiopathogenesis, contamination-free sampling is essential. Of course, microbiological results should always be assessed in the context of clinical signs. However the species should not be thoughtlessly dismissed as a contaminant, due to its histotoxic potential in a phase of highest susceptibility. It cannot be excluded that other virulence factors than those investigated in this work might play a role in the pathogenesis of uterine infections. The results of this work form the basis for further investigations to clarify the role of *C. perfringens* in the pathogenesis of typical disorders of the reproductive tract during puerperium that might enable us to better treat uterine disease. Finally, suitable hygiene management during the period of birth and intensive puerperal control are of critical importance in order to avoid infections and thus create good conditions for a new gravidity.

## Figures and Tables

**Table 1:** Classification of *C. perfringens* into five toxin types and the associated major toxins; + = major toxin present; − = major toxin not present, as described by Markey et al. [1].

*C. perfringens*	Major Toxin			
type	α	β	ε	ι
A	+	−	−	−
B	+	+	(+)	−
C	+	+	−	−
D	+	−	+	−
E	+	−	−	+

α = alpha-toxin, β = beta-toxin, ε = epsilon-toxin, ι = iota-toxin.

**Table 2:** Oligonucleotide primers for *C. perfringens* toxin gene detection.

Toxin/Gene	Primer	Oligonucleotide Sequence	Reference
alpha/*cpa*	CPALPHATOX-L CPALPHATOX-R	5′-AAGATTTGTAAGGCGCTT-3′ 5′-ATTTCCTGAAATCCACTC-3′	Buogo et al. [20]
beta/*cpb1*	CPBETATOX-L CPBETATOX-R	5′-AGGAGGTTTTTTTATGAAG-3′ 5′-TCTAAATAGCTGTTACTTTGTG-3′	Buogo et al. [20]
iota/*iap*	CPIOTA-L CPIOTA-R	5′-AATGCCATATCAAAAAATAA-3′ 5′-TTAGCAAATGCACTCATATT-3′	Braun et al. [21]
beta2/*cpb2*	P319BETA2 P320BETA2	5′-GAAAGGTAATGGAGAATTATCTTAATGC-3′ 5′-GCAGAATCAGGATTTTGACCATATACC-3′	Herholz et al. [22]

**Table 3:** Distribution of *C. perfringens*- positive diseased cows with respect to the disease patterns shown during puerperium. Numbers add up to more than 14, since some cows had more than one diagnosis.

Diagnosis	%	*n*
Retained fetal membranes	50.0	7
Puerperal metritis	21.4	3
Grade 1	14.3	2
Grade 2	7.1	1
Clinical metritis	92.9	13
Clinical endometritis	85.7	12
Urovagina	7.1	1

**Table 4:** Overview of the incidence of *C. perfringens* positive cows over the sampling period and localization.

Cows with a Pathological Puerperium (of Total 46)			Isolates
	%	*n*	*n*
	58.7	27	
*C. perfringens*-positive cows	51.9	14	21
localization of *C. perfringens*	%	*n*	
vagina	14.3	2	8
uterus	50.0	7	13
vagina and uterus	35.7	5	/ ^a^
time of sampling	%	*n*	
2	78.6	11 # *	15
4	21.4	3 #	3 #
6	21.4	3	3
14	0	0	0

# = 3 cows were positive on day 2 and 4 p.p.; fishers exact test: * *p* < 0.007. ^a^ = the five cases where cows were positive both in the uterus and the vagina resulted in 6 isolates from the vagina and 6 isolates from the uterus. These isolates are included in the respective lines for vagina and uterus.

**Table 5:** Minimum inhibitory concentration (MIC) range, MIC_50_, MIC_90_, CLSI- breakpoint values (mg/L) and prevalence (%) of antibiotic resistance in 21 *C. perfringens* isolates.

Antibiotics	MIC Range	MIC50	MIC90	CLSI-Breakpoints	Number of Resistant Isolates	Percentage of Resistant Isolates
**Penicillins/Beta-lactamase inhibitor**						
Penicillin G	0.0625–8	≤0.0625	0.125	S ≤ 0.5; R ≥ 2	2/21	9.5
Ampicillin	0.0625–8	≤0.0625	≤0.0625	S ≤ 0.5; R ≥ 2	1/21	4.8
Amoxicillin/Clavulanate	0.5/0.25–64/32	≤0.5/0.25	≤0.5/0.25	S ≤ 4/2; R ≥ 16/8	0/21	0
Piperacillin/Tazobactam	1/4–64/4	≤1/4	≤1/4	S ≤ 16/4; R ≥ 128/4	0/21	0
**Carbapenems**						
Meropenem	0.5–64	≤0.5	≤0.5	S ≤ 4; R ≥ 16	0/21	0
Imipenem	0.5–64	≤0.5	≤0.5	S ≤ 4; R ≥ 16	0/21	0
Ertapenem	0.125–16	≤0.125	≤0.125	S ≤ 4; R ≥ 16	0/21	0
**Nitromidazole**						
Metronidazole	0.25–32	1	2	S ≤ 8; R ≥ 32	1/21	4.8
**Fluoroquinolones**						
Moxifloxacine	0.0625–8	0.5	0.5	S ≤ 2; R ≥ 8	0/21	0
**Lincosamides**						
Clindamycine	0.0625–8	0.5	2.0	S ≤ 2; R ≥ 8	2/21	9.5
**Tetracyclines**						
Doxycycline	0.125–16	1	2	S ≤ 4; R ≥ 16	0/21	0
Tigecycline	1–8	≤1	≤1	S ≤ 4; R ≥ 16	0/21	0
**Glycopeptides**						
Vancomycin	2.0–8.0	≤2.0	≤2.0	S ≤ 2; R ≥ 4 *	0/21	0

S = susceptible, R = resistant; * = EUCAST breakpoint (version 11.0; January 2021); bold = antibiotic classes.

**Table 6:** Overview of the phenotypic reaction profile of *C. perfringens* isolates to selected antibiotics.

Antibiotics	Number of *C. perfringens* Isolates			
	1 ^a^	1 ^#, a^	1 ^b^	18 ^##, c^
Penicillin G	R	R	S	S
Ampicillin	R	I	S	S
Amoxicillin/Clavulanate	S	S	S	S
Piperacillin/Tazobactam	S	S	S	S
Meropenem	S	S	S	S
Imipenem	S	S	S	S
Ertapenem	S	S	S	S
Metronidazole	S	S	R	S
Moxifloxacine	S	S	S	S
Clindamycine	R	R	S	S
Doxycycline	S	S	S	S
Tigecycline	S	S	S	S
Vancomycin ^i^	S	S	S	S

i = here, evaluation was carried out via the breakpoint according to EUCAST (status: January 2021); R = resistant (highlighted in orange), I = intermediate (highlighted in yellow); S = susceptible; # = day 4 p.p., ## = day 4 p.p. (*n* = 2) and day 6 p.p. (*n* = 3), all other isolates were collected on day 2 p.p.; ^a^ = treated with benzylpenicillin procaine, ^b^ = treated with benzylpenicillin procaine, tetracycline hydrochloride and penethamathydroiodid (due to additional mastitis), ^c^ = 14 isolates from treated cows and 4 isolates from untreated cows, underlined: follow-up isolate from day 2 to day 4 p.p.

**Table 7:** Profiles of the accompanying findings of the *C. perfringens* positive cows (*n* = 14) with potential uterine pathogens.

Bacterial Concomitant Findings	1	1	1	1	1	1	1	1	1	1	1	3
*Escherichia coli*												
*Trueperella pyogenes*												
*Bacteroides* spp.												
*Enterococcus* spp.												
*Proteus* spp.												
*Fusobacterium* spp.												

## Data Availability

The data presented in this study is available on reasonable request from the corresponding author. Data are not publicly available due to privacy reason for the farm involved.

## References

[1] Markey B.K., Cullinane A., Leonard F.C., Maguire D., Archambault M., Hewat C. (2013). Clostridium species. Clinical Veterinary Microbiology.

[2] Fohler S., Klein G., Hoedemaker M., Scheu T., Seyboldt C., Campe A., Jensen K.C., Abdulmawjood A. (2016). Diversity of *Clostridium perfringens* toxin-genotypes from dairy farms. BMC Microbiol..

[3] Uzal F.A., Freedman J.C., Shrestha A., Theoret J.R., Garcia J., Awad M.M., Adams V., Moore R.J., Rood J.I., McClane B.A. (2014). Towards an understanding of the role of *Clostridium perfringens* toxins in human and animal disease. Future Microbiol..

[4] Selbitz H.J., Truyen U., Valentin-Weigand P. (2015). Gattung Clostridium. Tiermedizinische Mikrobiologie, Infektions- und Seuchenlehre.

[5] Uzal F.A., McClane B.A. (2011). Recent progress in understanding the pathogenesis of *Clostridium perfringens* type C infections. Vet. Microbiol..

[6] Canard B., Cole S.T. (1989). Genome organization of the anaerobic pathogen *Clostridium perfringens*. Proc. Natl. Acad. Sci. USA.

[7] Messelhäußer U. (2013). Pathogene Mikroorganismen: Clostridium Perfringens.

[8] Uzal F.A., Vidal J.E., McClane B.A., Gurjar A.A. (2010). Clostridium Perfringens Toxins Involved in Mammalian Veterinary Diseases. Open Toxinology J..

[9] Niilo L. (1980). *Clostridium perfringens* in animal disease: A review of current knowledge. Can. Vet. J..

[10] Nagahama M., Hayashi S., Morimitsu S., Sakurai J. (2003). Biological activities and pore formation of *Clostridium perfringens* beta toxin in HL 60 cells. J. Biol. Chem..

[11] Fennessey C.M., Sheng J., Rubin D.H., McClain M.S. (2012). Oligomerization of *Clostridium perfringens* Epsilon Toxin Is Dependent upon Caveolins 1 and 2. PLoS ONE.

[12] Nagahama M., Umezaki M., Oda M., Kobayashi K., Tone S., Suda T., Ishidoh K., Sakurai J. (2011). Clostridium perfringens Iota-Toxin b Induces Rapid Cell Necrosis. Infect. Immun..

[13] Gibert M., Jolivet-Renaud C., Popoff M.R. (1997). Beta2 toxin, a novel toxin produced by *Clostridium perfringens*. Gene.

[14] Klein C., Wehrend A., Weiss R., Bostedt H. (2007). Putrid-ulcerative vestibulo-vaginitis and myometritis in gravid sheep, caused by *Clostridium perfringens* type A. Tierärztl Prax.

[15] Dunaiev Y.K., Hadzevych O.V., Dunaieva O.V. Prevalence and Etiological Role of *Clostridium Perfringens* Bacteria in Dairy Farms. Proceedings of the The world of science and innovation: Abstracts of I International Scientific and Practical Conference.

[16] Jeon S.J., Lima F.S., Vieira-Neto A., Machado V.S., Lima S.F., Bicalho R.C., Santos J.E.P., Galvao K.N. (2018). Shift of uterine microbiota associated with antibiotic treatment and cure of metritis in dairy cows. Vet. Microbiol..

[17] Nak Y., Dagalp S.B., Cetin C., Nak D., Alkan F., Borum E., Tuna B. (2011). Course and severity of postpartum metritis cases following antibiotic and PGF2alpha administration in postpartum metritis cows infected with bohv-4. Transbound. Emerg. Dis..

[18] Edwards R. (1997). Resistance to beta-lactam antibiotics in *Bacteroides* spp.. J. Med. Microbiol..

[19] Gkiourtzidis K., Frey J., Bourtzi-Hatzopoulou E., Iliadis N., Sarris K. (2001). PCR detection and prevalence of α-, β-, β2-, ε-, ι- and enterotoxin genes in *Clostridium perfringens* isolated from lambs with clostridial dysentery. Vet. Microbiol..

[20] Buogo C., Capaul S., Hani H., Frey J., Nicolet J. (1995). Diagnosis of *Clostridium perfringens* type C enteritis in pigs using a DNA amplification technique (PCR). Zent. Vet. B.

[21] Braun M., Herholz C., Straub R., Choisat B., Frey J., Nicolet J., Kuhnert P. (2000). Detection of the ADP-ribosyltransferase toxin gene (cdtA) and its activity in *Clostridium difficile* isolates from *Equidae*. FEMS Microbiol. Lett..

[22] Herholz C., Miserez R., Nicolet J., Frey J., Popoff M., Gibert M., Gerber H., Straub R. (1999). Prevalence of beta2-toxigenic *Clostridium perfringens* in horses with intestinal disorders. J. Clin. Microbiol..

[23] Uzal F.A., Songer J.G. (2008). Diagnosis of *Clostridium perfringens* intestinal infections in sheep and goats. J. Vet. Diagn. Investig..

[24] Osman K.M., E-EM I., Ezzeldeen N.A., Hussein H.M.G. (2009). Mastitis in dairy buffalo and cattle in Egypt due to *Clostridium perfringens*: Prevalence, incidence, risk factors and costs. Rev. Sci. Tech. Off. Int. Epiz..

[25] Dylewski J., Wiesenfeld H., Latour A. (1989). Postpartum uterine infection with *Clostridium perfringens*. Rev. Infect. Dis..

[26] Halpin T.F., Molinari J.A. (2001). Diagnosis and Management of *Clostridium Perfringens* Sepsis and Uterine Gas Gangrene. Obstet. Gynecol. Surv..

[27] Kremer K.M., McDonald M.E., Goodheart M.J. (2017). Uterine *Clostridium perfringens* infection related to gynecologic malignancy. Gynecol. Oncol. Rep..

[28] Montavon C., Krause E., Holzgreve W., Hösli I. (2005). Uterine Gas Gangrene through *Clostridium Perfringens* Sepsis after Uterus Rupture Postpartum. Z. Geburtsh. Neonatol..

[29] Wang J., Sun C., Liu C., Yang Y., Lu W. (2016). Comparison of vaginal microbial community structure in healthy and endometritis dairy cows by PCR-DGGE and real-time PCR. Anaerobe.

[30] Williams E.J., Fischer D.P., Pfeiffer D.U., England G.C., Noakes D.E., Dobson H., Sheldon I.M. (2005). Clinical evaluation of postpartum vaginal mucus reflects uterine bacterial infection and the immune response in cattle. Theriogenology.

[31] Hadimli H.H., Erganis O., Sayin Z., Aras Z. (2011). Toxinotyping of *Clostridium perfringens* isolates by ELISA and PCR from lambs suspected of enterotoxemia. Turk. J. Vet. Anim. Sci..

[32] Parreira V.R., Russell K., Athanasiadou S., Prescott J.F. (2016). Comparative transcriptome analysis by RNAseq of necrotic enteritis *Clostridium perfringens* during in vivo colonization and in vitro conditions. BMC Microbiol..

[33] Guo S., Liu D., Zhang B., Li Z., Li Y., Ding B., Guo Y. (2017). Two Lactobacillus Species Inhibit the Growth and alpha-Toxin Production of *Clostridium perfringens* and Induced Proinflammatory Factors in Chicken Intestinal Epithelial Cells in Vitro. Front. Microbiol..

[34] Giguére S., Prescott J.F., Dowling P.M., Giguére S., Prescott J.F., Dowling P.M. (2013). Antimicrobial Therapy in Veterinary Medicine.

[35] Osman K.M., Elhariri M. (2013). Antibiotic resistance of *Clostridium perfringens* isolates from broiler chickens in Egypt. Rev. Sci. Tech..

[36] Agyare C., Boamah V.E., Zumbi C.N., Osei F.B., Kumar Y. (2018). Antibiotic use in poultry production and its effects on bacterial resistance. Antimicrobial Resistance: A global Threat.

